# Influence of Oxygen on Wound Healing Dynamics: Assessment in a Novel Wound Mouse Model under a Variable Oxygen Environment

**DOI:** 10.1371/journal.pone.0050212

**Published:** 2012-11-28

**Authors:** Hitomi Sano, Shigeru Ichioka, Naomi Sekiya

**Affiliations:** 1 Department of Surgical Science, Graduate School of Medicine, The University of Tokyo, Tokyo, Japan; 2 Department of Plastic and Reconstructive Surgery, Saitama Medical University Hospital, Saitama, Japan; University Hospital Hamburg-Eppendorf, Germany

## Abstract

**Introduction:**

Although oxygen is essential for the wound healing process, tissue hypoxia is known to stimulate angiogenesis. To explore these inconsistent findings, we estimated the influence of the oxygen environment on wound healing with our original model.

**Methods:**

Experiment 1 (Establishment of the model): To modify the topical oxygen tension, oxygen impermeable (polyvinylidene chloride) and permeable (polymethylpentene) membranes were applied to symmetrical excisional wounds in ddy mice (n = 6). Oxygen tension under the membrane was quantified with a device using photo-quenching technique. Experiment 2 (Influence of oxygen environment on wound healing): The wound area, granulation thickness and vascular density were analyzed under different oxygen environments (n = 24).

**Results:**

Experiment 1: The permeable group maintained equivalent oxygen level to atmosphere (114.1±29.8 mmHg on day 7), while the impermeable group showed extremely low oxygen tension (5.72±2.99 mmHg on day 7). Accordingly, each group was defined as the normoxia group and the hypoxia group. Experiment 2: Percent decrease in wound size was significantly enhanced in the normoxia group (11.1±1.66% on day 7) in comparison with the hypoxia group (27.6±3.47% on day 7). The normoxia group showed significantly thicker granulation tissue than the hypoxia group (491.8±243.2 vs. 295.3±180.9 µm). Contrarily, the vascular density of the hypoxia group significantly increased on day 7 (0.046±0.025 vs. 0.011±0.008 mm^2^/mm^2^).

**Conclusions:**

Our original model successfully controlled local oxygen concentration around the wound, and the hypoxic wounds showed increased angiogenesis but with a smaller amount of granulation tissue and delayed wound closure. Enhanced neovascularization in the hypoxic group likely implies compensative response to an insufficient ambient oxygen supply.

## Introduction

Oxygen is obviously essential to generate energy for the wound healing process including the metabolism, matrix synthesis, and cell migration and proliferation. Systemic as well as local oxygen supplementation has been reported to promote wound healing [1]–[4]. On the other hand, previous reports have suggested that tissue hypoxia stimulates angiogenesis during wound healing [5]. Hypoxia has also been reported to enhance angiogenic cytokines [6].

These contrary findings occasionally confuse clinicians and researchers pursing the best wound treatment. Despite the evident significance of oxygen in wound healing, the optimal oxygen environment during repair process remains unclear. This confusion may mainly results from lack of an appropriate experimental model which can estimate wound conditions under different topical oxygen environments. We hypothesized that occlusion of the wound by membranes with different oxygen permeability could create a different oxygen environment at the wound surface. However, the major problem included the difficulty in quantification of oxygen tension (PO_2_) around the wounds.

To address the latter problem, our research group has developed an original topical oxygen tension measurement system using O_2_-dependent quenching of the phosphorescence technique, which allows regional determination of intravascular and interstitial PO_2_ values [7]–[11]. The basis of this technique is a well-known reaction in which Pd-porphyrin is excited to its triplet state by exposure to pulsed light, after which phosphorescence intensity is reduced by emission and energy transfer to O_2_. Oxygen tensions values around the pd-porphyrin can be calculated by measuring the reflected light intensity [7].

We have considered that our oxygen measurement technique might be applicable to evaluation of the oxygen environment around a wound, and sought to establish an original methodology. During our trial we encountered a fine oxygen measurement device (Fibox 3®, PreSens), using the same photo-quenching principle. Employment of this device allowed the innovative experimental model in the current study. The present study aimed at introducing our novel model and to elucidate the influence of the oxygen environment on wound healing.

## Materials and Methods

### Ethics Statement

All the procedures were performed according to the guidelines of the Animal Care and Use Committee of Saitama Medical University. The protocol was approved by the committee on the Ethics of Animal Experiment of Saitama Medical University (permit number: 454).

Firstly, a wound healing model was established in which the oxygen condition around the wound could be intentionally changed, and we assessed its validity in experiment 1. Subsequently, the wound healing dynamics under different oxygen environments were quantitatively assessed in experiment 2.

### Animals and Wound Creation

Ddy mice, aged 7 to 9 weeks were obtained from Crea Japan Inc. (Tokyo, Japan). The body hair of the mice was depilated using hair removal cream and the mice were anesthetized using isoflurane inhalation prior to the following surgical procedures. Two symmetrical full-thickness skin wounds (diameter = 8 mm) were made on each side of each mouse’s back using microforceps and scissors under an operating microscope.

### Experiment 1: Development and Assessment of a New Wound Model under Variable Topical Oxygen Environment

#### Development of a wound model under variable oxygen environment

**Figure 1:**
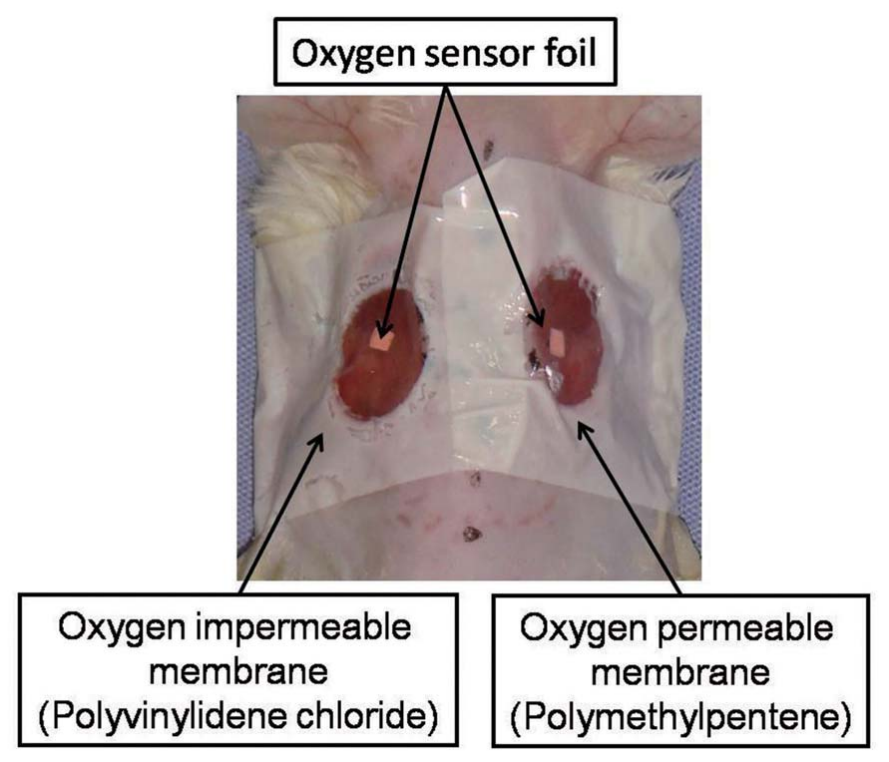
Mouse model. In order to establish different oxygen environments, the wound on one side of the dorsum was covered with an oxygen permeable membrane, and an oxygen impermeable membrane was applied to the wound on the other side. Oxygen sensor foils were attached under the membranes.

In order to continuously control the topical oxygen tension around the wound, the wound on one side was sealed up with an oxygen permeable membrane (n = 6). The contralateral wound was covered with an oxygen impermeable membrane. Polymethylpentene (Rikentechnos, Tokyo, Japan) and polyvinylidene chloride (Saran Wrap®, Asahikasei, Tokyo, Japan) were respectively employed as the oxygen permeable and oxygen impermeable membranes. The oxygen permeability of each membrane was 60000–65000 and 40–90 ml/m^2^*24 hr*1 atm, respectively. Quantitative data of the manufactures (Rikentechnos and Asahikasei) supplying membranes showed that both have compatible transparency with opacity 0.3%. Both membranes are commercially produced as kitchen wrap. Two manufactures verified that films contained no substances listed on the “Strategic Programs on Environmental Endocrine Disruptors ‘98” (SPEED ‘98) program, including phthalic acid ester, adipic acid ester, and nonyl phenol. To fix the membranes to the skin around the wounds, adhesive tapes with a hole of a diameter of 1 cm were attached to the membranes (Figure 1). The groups were defined as the permeable group and the impermeable group.

#### Assessment of oxygen tension around the wound

**Figure 2:**
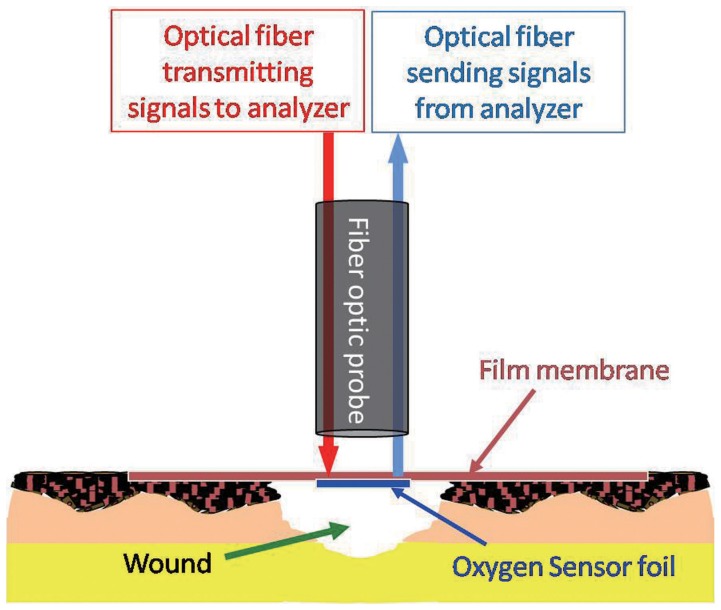
Measurement of topical oxygen tension. The transparent membranes allow measurement of the topical oxygen tension around the oxygen sensor, namely the oxygen environment which is directly in contact with the wound, by perceiving the reflected light intensity of the pulsed light which exogenously irradiated the sensor via the membrane.

A Fibox 3® oxygen meter (Fibox 3®, PreSens, Germany), whose principle is based on the technique of O_2_-dependent quenching of phosphorescence, was used in this study. The system consisted of a fiber optic probe and a trace oxygen sensor foil, and enabled oxygen measurement in a non-destructive way. In our pilot study we have confirmed that various oxygen tensions measured by the device (Fibox 3®, PreSens) through each membrane indicated no difference from the values obtained by direct measurement without membrane. Intervention of the membranes between fiber optic probe and sensor foil did not affect the measured data. An oxygen sensor foil of 2×2 mm^2^ was attached to the wound side of the membrane (Figure 1 and 2). The transparent membrane allowed measurement of the topical oxygen tension around the oxygen sensor, namely the oxygen environment which was directly contact with the wound, by perceiving the reflected light intensity of the pulsed light which exogenously irradiated the sensor via the membrane (Figure 1 and 2).

### Experiment 2: Quantification of Wound Healing under Different Oxygen Environments

This experiment aimed to evaluate the wound healing dynamics under the different oxygen environments using the model (n = 24) established in experiment 1. The wound area on days 1, 3, 5 and 7, and granulation thickness and vascular density on day 7 were analyzed.

#### Assessment of the wound area

For a macroscopic evaluation of wound healing, each wound edge was traced and the area was measured digitally using Scion Image software for Windows (beta 4.02 version; Scion Corporation, Frederick, MD, USA). We defined the percentage decrease in the wound area using the following equation: Percentage decrease in the wound area (%) = (wound area on each day/wound area on operative day) × 100 (%).

#### Histological and Immunohistochemical Evaluation

The animals were sacrificed on day 7. The wound beds on both sides were harvested together with a surrounding margin of normal skin and the underlying muscle layer. Each tissue specimen was fixed with zinc-fixative solution (IHC ZINC fixative, BD Pharmingen, Tokyo, Japan) and embedded in paraffin. Hematoxylin and eosin (H&E) staining was used to detect the thickness of the granulation. Immunohistochemical staining for CD34 was conducted to visualize the blood vessels. Deparaffinized thin sections were stained with anti-mouse CD34 (BD Pharmingen, Tokyo, Japan) and visualized using a horseradish peroxidase/diaminobenzidine (DAB) substrate kit (BD Pharmingen, Tokyo, Japan).

The mean length from the wound surface to the granulation base of the right and left ends of the wound and central part were calculated as the granulation thickness. The density of CD34 positive structures was measured using image processing and analyzing software. The density was calculated as the area of the positive structures relative to the total area and defined as vascular density (mm^2^/mm^2^). The digitized microscopic images were montaged using Win Roof version 5.5 (Mitani Corporation, Japan) software. The intravascular area in the montaged image was colored to demarcate the vessels, and the background tissue was erased using image processing software (Adobe Photoshop, San Jose, CA, USA). To enumerate the total area of the vessels in a section, each image was converted into binary format and processed using Scion Image 4.02 software. The total area of the given section was also measured digitally.

### Statistical Analysis

Data were expressed as the mean ± standard deviation. Differences between the groups were compared using a paired Student’s *t*-test. To detect differences between the two groups, an analysis of covariance was employed. All statistical analyses were performed using SPSS 15.0J (IBM, USA). Values of p<0.05 were considered significant.

## Results

### Experiment 1

**Figure 3:**
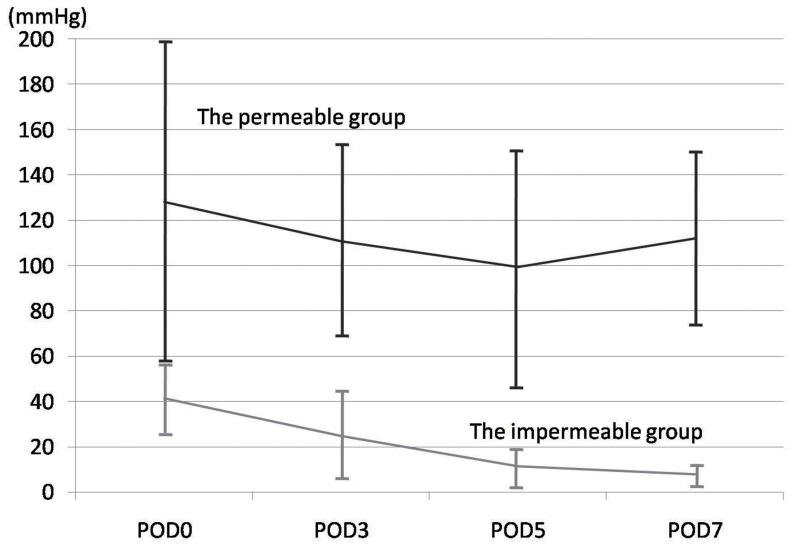
Time course of the local oxygen tensions. The oxygen tensions (mmHg) on day 0 (6–8 hours after wound creation), 3, 5, and 7 were serially assessed. Means with SD are presented. The permeable group maintained an oxygen level equivalent to the atmosphere, whereas the impermeable group showed an extremely low oxygen tension.

The local oxygen tensions of the wound surfaces under the membrane were serially assessed. The permeable group maintained an oxygen level equivalent to the atmosphere (approximately 150 mmHg), whereas the impermeable group showed extremely low oxygen tension. The oxygen tensions (mmHg) on day 0 (6–8 hours after wound creation), 3, 5, and 7 of the impermeable group were 39.7±15.8, 21.8±16.03, 8.25±4.45, 5.72±2.99 mmHg, and those of the permeable group were 119.3±73.5, 111.97±24.6, 102.5±46.3, 114.1±29.8 mmHg, respectively) (Figure 3). The between groups test indicated that the difference between the groups was significant (p<0.01). The within subject test indicated that there was not a significant time effect (p<0.01). Accordingly, the permeable and the impermeable groups were respectively defined as the normoxia group and the hypoxia group.

### Experiment 2

**Figure 4:**
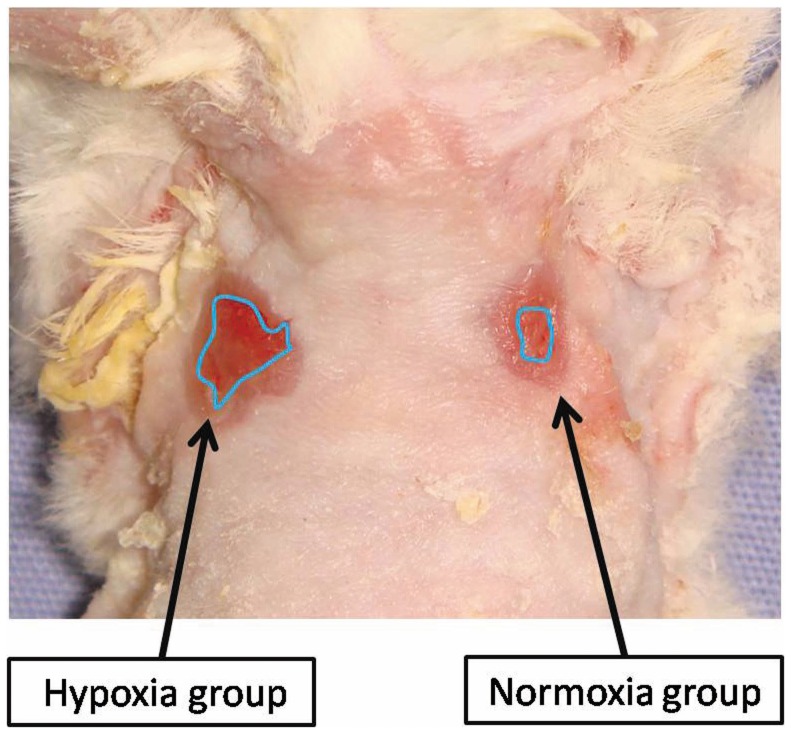
Macroscopic finding on day 7. Wound size significantly decreased in the normoxia group in comparison with the hypoxia group.

**Figure 5:**
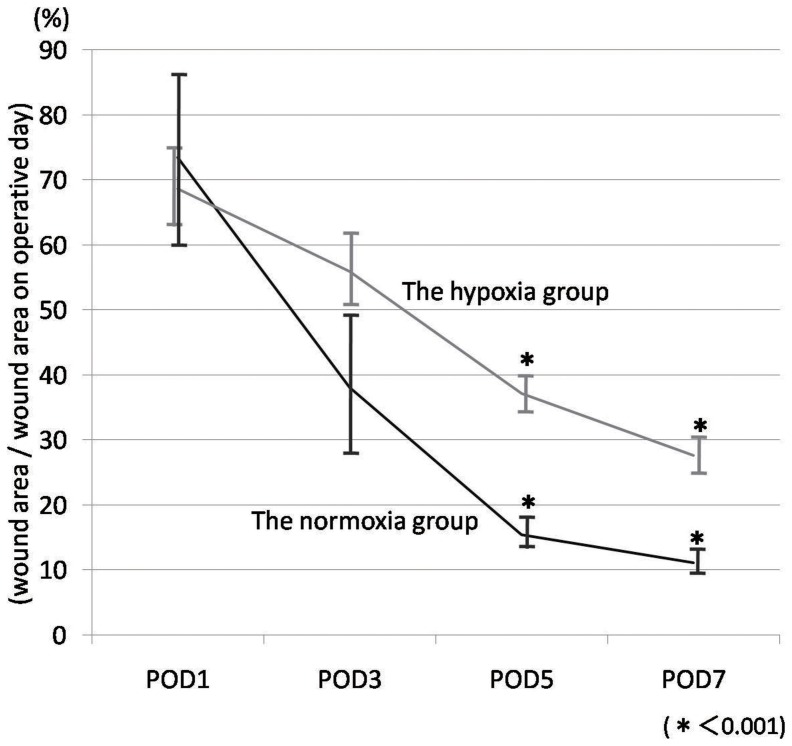
Time course of the wound contraction rates. Wound size significantly decreased in the normoxia group in comparison with the hypoxia group on day 5 and 7. The interaction of time and group is significant which means that the groups were changing over time but were changing in different ways.

The representative macroscopic appearance on day 7 is shown in Figure 4. Wound size significantly decreased in the normoxia group in comparison with the hypoxia group on day 5, and 7. The wound contraction rates (wound area/wound area on the operative day as a percentage) on days 1, 3, 5, and 7 of the hypoxia group were 68.6±8.28, 56.0±6.41, 37.1±3.45, and 27.6±3.47%; and those of the normoxia group were 73.5±14.3, 38.1±10.8, 15.4±2.03, and 11.1±1.66%, respectively (p<0.01) (Figure 5). The interaction of time and group was significant which means that the groups were changing over time but were changing in different ways (p<0.01) (Figure 5).

**Figure 6:**
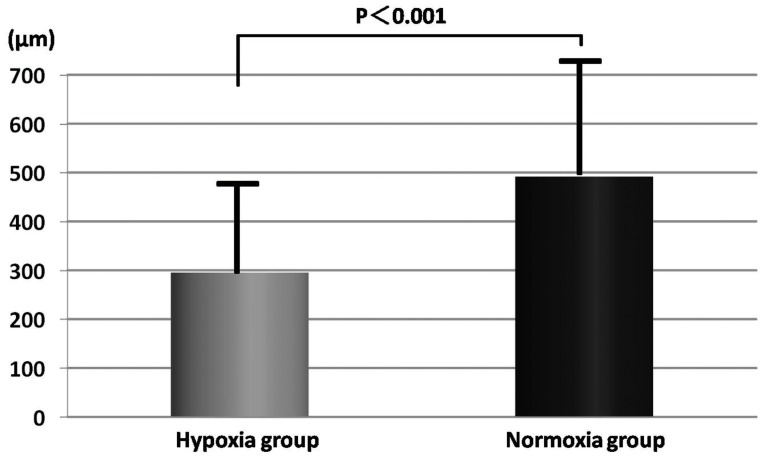
Granulation tissue thickness on day 7. The granulation was significantly thicker in the normoxia group in comparison with the hypoxia group. The mean granulation thickness in each section of the normoxia group was 491.8±243.2 µm. In contrast, the mean thickness of the hypoxia group was 295.3±180.9 µm.

**Figure 7:**
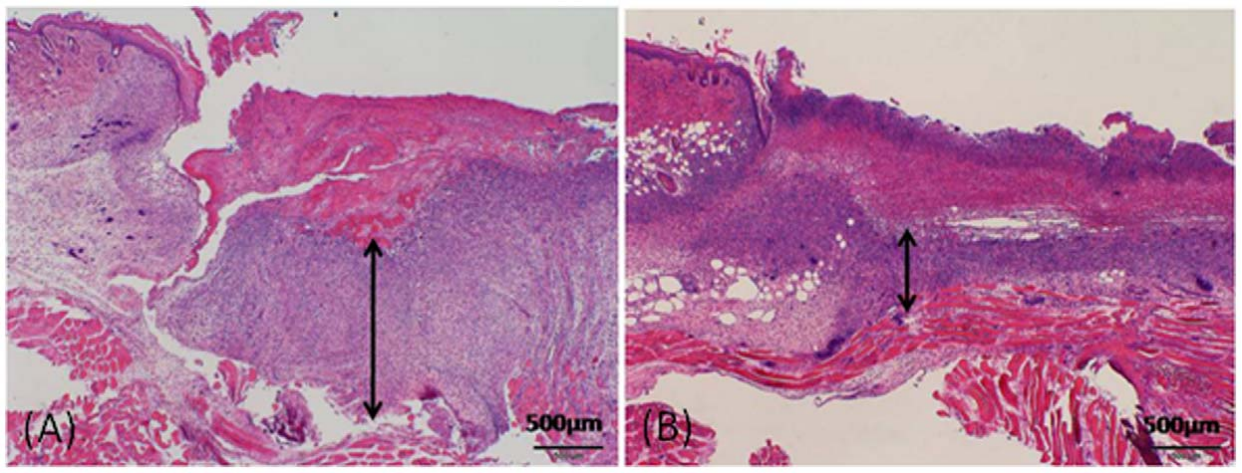
Microscopic images of granulation tissue. Tissue preparations were obtained on day 7. The thickness of granulation tissue (double-headed arrows) significantly increased in the normoxia group (A) as compared to the hypoxia group (B).

**Figure 8:**
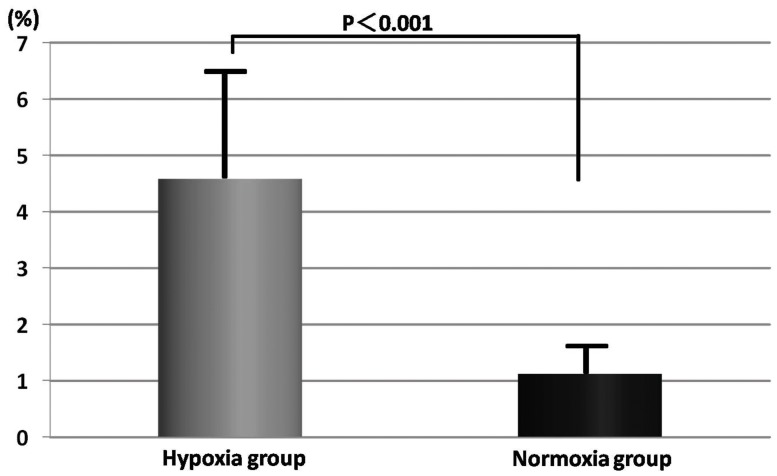
Vascular density on day 7. The vascular density of the hypoxia group significantly increased. The mean vascular density in each section of the normoxia group was 0.011±0.008 mm^2^/mm^ 2^. In contrast, the mean thickness of the hypoxia group was 0.046±0.025 mm^2^/mm^ 2^.

**Figure 9:**
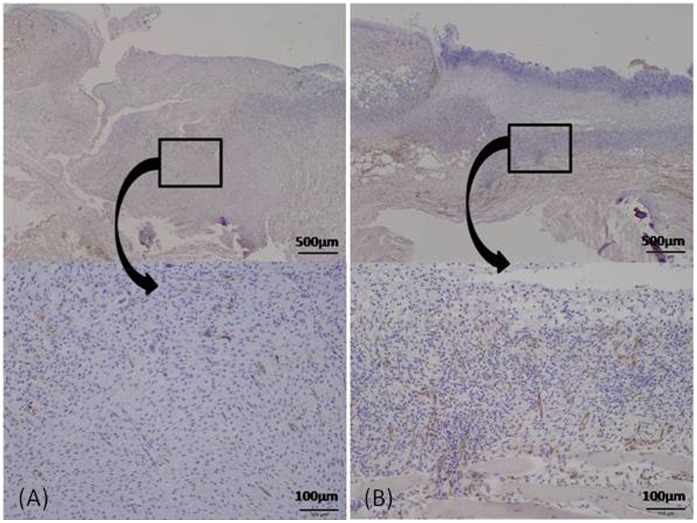
Histological evaluation for vasculature. Tissue preparations were obtained on day 7. The immunostained section revealed that the vascular density of the hypoxia group (B) was significantly higher than that of the normoxia group (A).

The granulation thickness and the vascular density of each group were compared on day 7. The granulation was significantly thicker in the normoxia group in comparison with the hypoxia group (491.8±243.2 vs. 295.3±180.9 µm, p<0.01) (Figures 6 and 7). The vascular density of the hypoxia group significantly increased in comparison with the normoxia group (0.046±0.025 vs. 0.011±0.008 mm^2^/mm^ 2^, p<0.01) (Figures 8 and Figure 9).

## Discussion

In order to assess the influence of oxygen on wound healing, most of the previous studies have placed cells or animals in various oxygen environments without measurement of the oxygen tension [1], [2], [4], [12]–[15]. However, these experimental approaches provide only partial information. In contrast, our experimental model offered the comprehensive effect of topical oxygen on wound healing dynamics. The model successfully controlled the local oxygen concentration as the microclimate of the wound in the same animal. Application of the advanced optical oxygen sensor to the oxygen changeable wound model enabled simultaneous analysis of oxygen tension and wound healing parameters.

Oxygen plays an essential role in wound healing and is involved in multiple wound healing processes including reepithelialization and collagen synthesis [1]–[4], [16]. Recent reports have showed that superficial layers of the skin are oxygenated via diffusion from the ambient air [1], [17]–[18]. The stratum corneum in intact skin represents a significant barrier for diffusion from the atmosphere [17]. Once the stratum corneum is broken, oxygen diffusion increases [17]–[18]. When the outer layers are disrupted in wounds, oxygen can diffuse into the deeper layers [1]. Thus, the oxygen supply from both the atmosphere and the vessels has an effect on the wound healing process.

Several studies have suggested that oxygen promotes epithelialization [1], [2]. Topical oxygen accelerated wound closure in full thickness excisional wounds in the pig model [1]. Transdermal oxygen improved epithelial healing in a rabbit ear ischemic wound model [2].

Granulation tissue consists of new vessels, cells and extracellular matrix (ECM) components. Collagen is the main protein of the ECM and the most abundant protein, making up about 30% of the whole-body protein content. Synthesis and deposition of collagen require large amounts of oxygen [3], [4]. Niinikoski et al. showed that wound tensile strength rose 25% in 50% oxygen compared to atmospheric pressure in rats [3]. Increasing wound oxygenation resulted in increased collagen deposition and tensile strength in rabbits [4]. Postoperative patients treated with supplemental oxygen produced 3 times as much collagen when wounds were perfused and oxygenated compared to patients with lower perfusion [16].Conversely, a couple of previous studies demonstrated that hypoxia enhances angiogenesis [5], [15]. Knighton et al. suggested that a hypoxic tissue gradient is mandatory for wound healing-related angiogenesis using the rabbit ear wound model [5]. Cherry and Ryan reported that a hydrocolloid dressing accelerated angiogenesis due to a similar hypoxic effect [15]. Some other studies experimentally suggested that an oxygen impermeable dressing promoted neovascularization using normal wound healing animal models, and concluded that the oxygen impermeable dressing might be preferable for clinical use [5], [15].

Thus, some studies indicated sufficient oxygen supply promoted wound healing while others suggested hypoxia enhanced wound healing angiogenesis. This evokes a fundamental question “What is the optimal oxygen environment for wound healing?” Our findings provide a hint to answer the question. In the present study hypoxic wounds showed increased angiogenesis with less amount of granulation tissue and delayed wound closure. The results were consistent with the previous contradictory knowledge. One possible explanation for this paradox is that enhanced neovascularization in the hypoxic group implied a compensative response to an insufficient ambient oxygen supply. Although hypoxia induces angiogenesis, intentional blockade of air diffusion to wounds with oxygen impermeable dressings may not be an advisable strategy. Our findings suggest that an adequate ambient oxygen supply was preferable for eventual wound healing since all the activities of organisms require oxygen.

### Conclusions

Our novel wound model described herein allowed for study of the whole wound healing dynamics under variable oxygen environments. Wound size significantly decreased and granulation was thicker in the normoxia group in comparison with the hypoxia group. The vascular density of the hypoxia group significantly increased. Our result suggested that sufficient oxygen allowed adequate epithelialization and granulation formation. Enhanced neovascularization in the hypoxia group likely implied compensation for the hypoxic condition.
